# Maternal exposure to intimate partner violence and breastfeeding practices in 51 low-income and middle-income countries: A population-based cross-sectional study

**DOI:** 10.1371/journal.pmed.1002921

**Published:** 2019-10-01

**Authors:** Rishi Caleyachetty, Olalekan A. Uthman, Hana Nekatebeb Bekele, Rocio Martín-Cañavate, Debbie Marais, Jennifer Coles, Briony Steele, Ricardo Uauy, Peggy Koniz-Booher

**Affiliations:** 1 Warwick Medical School, University of Warwick, Coventry, United Kingdom; 2 World Health Organization Inter-Country Support Team, Zimbabwe WHO Country Office, Harare, Zimbabwe; 3 National Centre of Tropical Medicine, Carlos III Institute of Health, Madrid, Spain; 4 School of Medicine, Pontificia Universidad Católica de Chile, Santiago, Chile; 5 JSI Research & Training Institute, Arlington, Virginia, United States of America; London School of Hygiene and Tropical Medicine, UNITED KINGDOM

## Abstract

**Background:**

Intimate partner violence (IPV) against women is a major global health issue, particularly in low- and middle-income countries (LMICs), that is associated with poor physical and mental health, but its association with breastfeeding practices is understudied. Both the World Health Organization (WHO) and the United Nations Children’s Fund (UNICEF) recommend that children initiate breastfeeding within the first hour of birth and be exclusively breastfed for the first 6 months of life. Breastfeeding within the first hour of birth is critical to newborn survival, and exclusive breastfeeding for 6 months is recognised to offer significant health benefits to mothers and their infants. We examined the association of maternal exposure to IPV with early initiation of breastfeeding (within 1 hour of birth) and exclusive breastfeeding in the first 6 months.

**Methods and findings:**

We assessed population-based cross-sectional Demographic and Health Surveys (DHS) from 51 LMICs. Data from the most recent DHS in each country (conducted between January 2000 and January 2019) with data available on IPV and breastfeeding practices were used. By WHO region, 52.9% (27/51) were from Africa, 11.8% (6/51) from the Americas, 7.8% (4/51) from the Eastern Mediterranean, 11.8% (6/51) from Europe, 11.8% (6/51) from South-East Asia, and 3.9% (2/51) from the Western Pacific. We estimated multilevel logistic regression models for any IPV and each type of IPV separately (physical violence, sexual violence, and emotional violence), accounting for demographic and socioeconomic factors. Depending on specification, the sample size varied between 95,320 and 102,318 mother–infant dyads. The mean age of mothers was 27.5 years, and the prevalence of any lifetime exposure to IPV among mothers was 33.3% (27.6% for physical violence, 8.4% for sexual violence, and 16.4% for emotional violence). Mothers exposed to any IPV were less likely to initiate breastfeeding early (adjusted odds ratio [AOR]: 0.88 [95% CI 0.85–0.97], *p* < 0.001) and breastfeed exclusively in the first 6 months (AOR: 0.87 [95% CI 0.82–0.92], *p* < 0.001). The associations were similar for each type of IPV and were overall consistent across infant’s sex and WHO regions. After simultaneously adjusting for all 3 types of IPV, all 3 types of IPV were independently associated with decreased likelihood of early breastfeeding initiation, but only exposure to physical violence was independently associated with a decreased likelihood of exclusively breastfeeding in the first 6 months. The main limitations of this study included the use of cross-sectional datasets, the possibility of residual confounding of the observed associations by household wealth, and the possibility of underreporting of IPV experiences attenuating the magnitude of observed associations.

**Conclusions:**

Our study indicates that mothers exposed to any form of IPV (physical, sexual, or emotional violence) were less likely to initiate breastfeeding early and breastfeed exclusively in the first 6 months. These findings may inform the argument for antenatal screening for IPV in LMICs and the provision of services to not only improve mothers’ safety and well-being, but also support them in adopting recommended breastfeeding practices.

## Abstract 2

Rishi Caleyachetty reveal that women exposed to intimate partner violence are less likely to initiate breastfeeding of newborns.

## Author summary

**Why was this study done?:**

- Intimate partner violence is common in low- and middle-income countries and may affect women’s choice or ability to breastfeed.
- We sought to examine the association between mothers’ exposure to intimate partner violence and breastfeeding practices (early initiation of breastfeeding and exclusive breastfeeding in the first 6 months).

**What did the researchers do and find?:**

- We used nationally representative survey data from 51 low- and middle-income countries conducted between January 2000 and January 2019.
- Mothers exposed to intimate partner violence were less likely to engage in recommended breastfeeding practices including initiating breastfeeding within the first hour of birth and breastfeeding exclusively in the first 6 months.

**What do these findings mean?:**

- In low- and middle-income countries, health professionals working with pregnant women may need to identify those who have experienced intimate partner violence and offer tailored support for breastfeeding practices.

## Introduction

Intimate partner violence (IPV) is a common and costly global health issue [1,2], particularly in low- and middle-income countries (LMICs). Globally, a third of women have been the victims of gender-based violence [3], with a higher risk of exposure during and after pregnancy [4].

The experience or fear of physical, sexual, or emotional violence within a relationship [5] can affect a mother’s physical health and mental health [6,7], which in turn condition a mother’s choice or ability to breastfeed. Breastfeeding improves the survival, health, and development of children, prevents breast cancer, and improves birth spacing [8]. As a result, the World Health Organization (WHO) recommends the early initiation of breastfeeding (within 1 hour after birth) and exclusive breastfeeding (no other food or drink, not even water) for at least 6 months [9].

Breastfeeding is a human rights issue for mothers and their children, and should be protected and promoted for the benefit of both [10]. A mother is not obligated to breastfeed her child, but no one should interfere with a mother’s right to breastfeed her child [11]. The Office of the United Nations High Commissioner for Human Rights declares that children have the right to life, survival, and development and to the highest attainable standard of health, of which breastfeeding must be viewed as an integral component [10]. In a recent systematic review of IPV and breastfeeding practices [12], only 12 studies examining the association between IPV and breastfeeding behaviours were identified. Of these, 8 reported lower breastfeeding intention, breastfeeding initiation, and exclusive breastfeeding during the first 6 months of the child’s life, and a higher likelihood of early termination of exclusive breastfeeding, among mothers who experienced IPV. Only 6 studies were from LMICs [13–18], and of these only 2 measured a mother’s exposure to emotional violence. Early initiation of breastfeeding (within 1 hour after birth) was measured in few studies [15,18]. Considering the dearth of studies examining maternal exposure to IPV and breastfeeding practices in LMICs, we examined the association of maternal exposure to IPV with early initiation of breastfeeding and exclusive breastfeeding in the first 6 months in 51 LMICs.

## Methods

Our prospective analysis plan is in S1 Text, and no data-driven changes have taken place.

### Study design and participants

Demographic and Health Surveys (DHS) [19] are population-based cross-sectional household surveys carried out at approximately 5-year intervals in a range of countries, mainly LMICs. We used data from the most recent DHS in each country (conducted between January 2000 and January 2019) with data available on IPV and breastfeeding practices (S1 Table). The datasets used for this project are available at the DHS Program website (https://www.dhsprogram.com/data/available-datasets.cfm). In all of the countries where the surveys were conducted, the researchers followed the same standardised procedures (survey instruments, interview training, data collection, and data processing). Complete descriptions of country DHS sampling, questionnaire validation, data-collection methods, and data-validation procedures are published elsewhere [19]. The DHS sampling frame is a list of enumeration areas that cover an entire country and serve as the clusters in the survey of that country. All households in the selected clusters are listed, and a fixed proportion is selected by systematic sampling. The DHS domestic violence module was administered to 1 randomly chosen woman per household in the age range of 15–49 years, who was matched to her child aged less than 24 months. Before participating, all women were asked to provide written informed consent. Ethical approval for the DHS was provided centrally by the ORC Macro Institutional Review Board and by individual review boards within each participating country.

### Breastfeeding practices

Two WHO-recommended indicators for assessing breastfeeding practices were included: early breastfeeding (the proportion of children born in the last 24 months who were put to the breast within 1 hour of birth) and exclusive breastfeeding in the first 6 months (the proportion of infants aged 0–5 months who are fed exclusively with breast milk). The time frame 0–5 months includes from birth through the end of the infant’s fifth month of life [9]. Early initiation of breastfeeding was assessed from the question ‘How long after birth did you first put (name of the child) to the breast?’ The answers were recorded in hours and days after birth. Infants under 24 months of age were categorised as being breastfed early if they were breastfed within 1 hour of birth. Exclusive breastfeeding was assessed from the question ‘Are you currently breastfeeding (name of the child)?’ A ‘yes’ response was followed by other questions on additional liquids or solid foods fed to the infant in the past 24 hours. Infants under 6 months of age were categorised as being exclusively breastfed at the time of interview if they were breastfed in the past 24 hours but did not receive any other types of food.

### Measure of maternal exposure to IPV

Exposure to IPV was measured by binary (i.e., yes or no) indicators of physical, sexual, and emotional violence. Information about IPV was collected from 1 randomly selected mother in each household, with no one else in the household aware that this was done. Married or cohabiting mothers were asked about ever having experienced IPV by their husband or partner, whereas formerly married or formerly cohabiting women were asked about IPV by their most recent husband or partner. The DHS domestic violence module is a modified and abbreviated version of the Revised Conflict Tactics Scales (CTS2) [20], in which the questions ask about specific acts.

Physical violence was assessed via 7 items and was indicated by a positive response to any one of the following experiences of behaviour from a woman’s husband or partner: ‘push you, shake you or throw something at you’, ‘slap you’, ‘twist your arm or pull your hair’, ‘punch you with his fist or with something that could hurt you’, ‘kick you, drag you or beat you up’, ‘try to choke you or burn you on purpose’, or ‘threaten or attack you with a knife, gun or any other weapon’.

Sexual violence was assessed via 2 items and was indicated by a positive response to either of the following experiences of behaviour from a woman’s husband or partner: ‘physically force you to have sexual intercourse with him even when you did not want to’ or ‘force you to perform any sexual acts you did not want to’.

Emotional violence was assessed via 2 items and was indicated by a positive response to either of the following experiences of behaviour from a woman’s husband or partner: ‘say or do something to humiliate you in front of others’ or ‘threaten to hurt or harm you or someone close to you’.

We created binary variables for lifetime exposure to physical, sexual, or emotional violence, and to each type of IPV separately.

### A priori confounding variables

Maternal age, level of education, household wealth, place of residence, and child’s age and sex were considered a priori confounding variables [15,18]. Maternal education was assessed by self-report of completed educational level (no education, primary, secondary, or higher). DHS measures household wealth as a composite measure of household assets (e.g., bicycles, cars, and radios) and characteristics (e.g., flooring material, drinking water source, and type of toilet facility). Household wealth is categorised as 5 quintiles (poorest, poor, middle, rich, or richest). The participants’ place of residence was categorised as rural versus urban. Child’s age was recorded in months and calculated from the child’s date of birth. Child sex was recorded as male or female.

### Statistical analysis

We pooled individual-level data from the nationally representative DHS to create a global sample that followed a 3-level hierarchical structure with mothers at level 1, nested within communities at level 2 and countries at level 3. To account for the complex survey design, we used multilevel logistic regression models to estimate adjusted odds ratios (AORs) and 95% confidence intervals (95% CIs) for the association of maternal exposure to IPV with early initiation of breastfeeding and exclusive breastfeeding. All models controlled for the same set of a priori confounding variables including mother’s age, mother’s level of education, household wealth, rural or urban residence, child’s age, and child’s sex. Models were run for each IPV type separately (physical violence, sexual violence, and emotional violence), any type of IPV, and then each IPV type while simultaneously adjusting for all 3 types of IPV.

To examine the consistency of the main findings among different subpopulations, we undertook a priori subgroup analysis defined by child sex and WHO region (Africa, Americas, Eastern Mediterranean, Europe, South-East Asia, and Western Pacific). We present subgroup-specific AORs and 95% CIs using multilevel logistic regression models. To test for differences in the associations between subgroups, *p*-values for interaction were derived by including an interaction term in the multilevel logistic regression model.

Data were not weighted, given that DHS weights are country-specific and DHS does not provide weights that are appropriate for multilevel analysis. Although not reported here, we also tested for a cross-level interaction effect between the household wealth index and country. We found that the effect of the wealth index varies by country; however, the effect of maternal exposure to IPV, our main exposure of interest, does not vary across countries. The magnitude and direction of the association of interest did not change when we examined the cross-level interaction.

Stata 14.0 (StataCorp, College Station, TX) was used for data preparation. MLwiN 3.00 was used to fit all multilevel models and estimated parameters using iterative generalised least squares or marginal quasi-likelihood algorithms.

## Results

Surveys were conducted in 69 countries after 2000; however, only 74.0% (51/69) had data on IPV and breastfeeding practices and were therefore included in our analysis (S1 Table). By WHO region, 52.9% (27/51) were from Africa, 11.8% (6/51) from the Americas, 7.8% (4/51) from the Eastern Mediterranean, 11.8% (6/51) from Europe, 11.8% (6/51) from South-East Asia, and 3.9% (2/51) from the Western Pacific.

**Fig 1:**
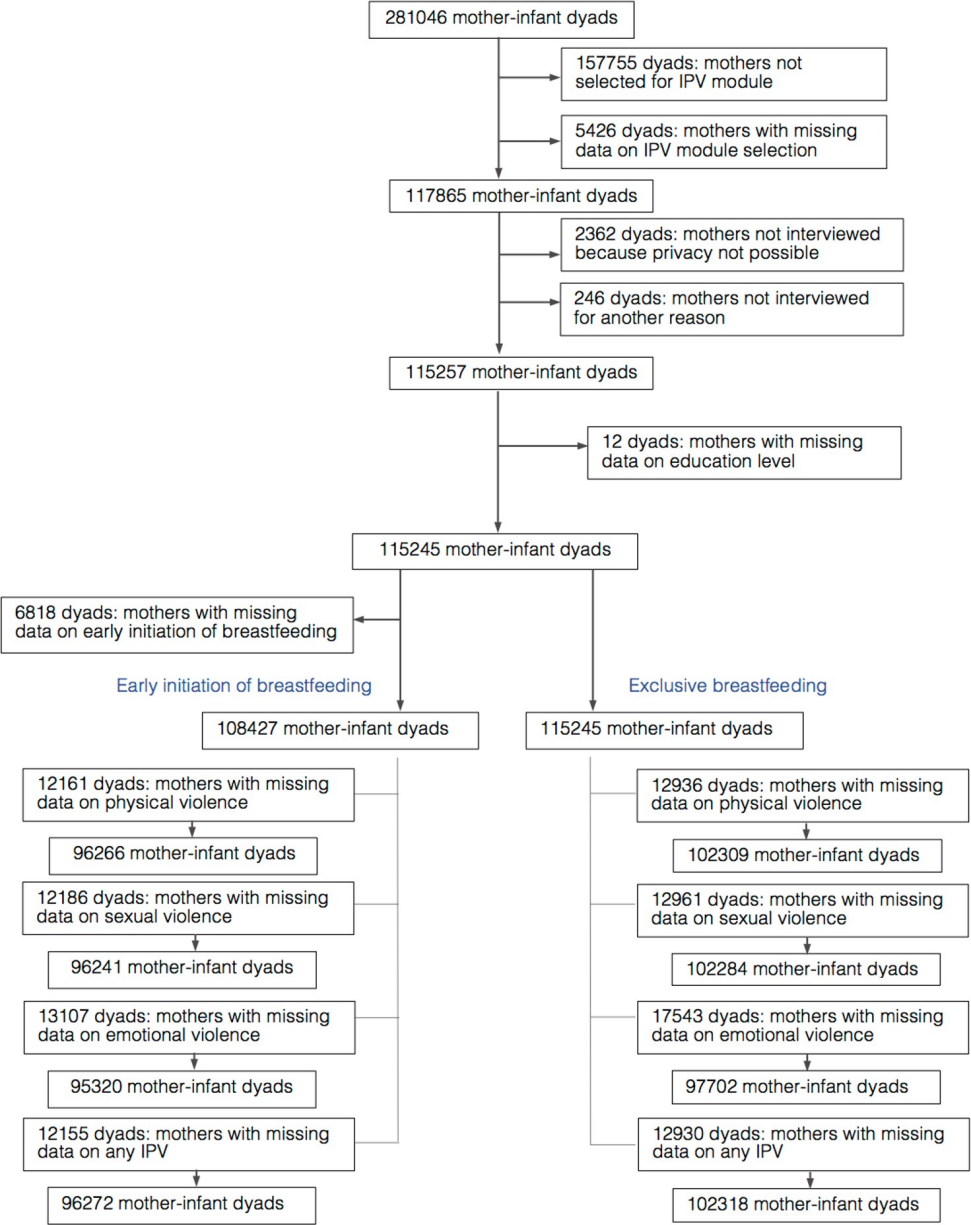
Sample selection. IPV, intimate partner violence.

The study population included mothers aged 15–49 years who were matched to their child aged less than 24 months, who had completed the IPV module, and who also had answered questions about breastfeeding their youngest child. A total of 281,046 mother–infant dyads were potentially eligible for inclusion in our sample based on the infant’s age at the time of the interview (Fig 1). Of these dyads, 117,865 mothers were randomly selected for the IPV module, but 2,362 of these mothers were excluded because the interview could not be conducted in private, another 246 because the mother could not be interviewed for another reason, and another 12 for missing data on education level, yielding a total sample of 115,245 mother–infant dyads. Other exclusions for missing data for early initiation of breastfeeding resulted in a sample of 108,427 for the early initiation of breastfeeding analysis (96,266 for physical violence, 96,241 for sexual violence, 95,320 for emotional violence, and 96,272 for any IPV) and 115,245 for the exclusive breastfeeding analysis (102,309 for physical violence; 102,284 for sexual violence; 97,702 for emotional violence, and 102,318 for any IPV).

Mothers who were selected for the IPV module but were excluded from this analysis because of missing data on early initiation of breastfeeding or IPV were less likely to initiate breastfeeding within 1 hour of birth (41.4% versus 46.2%, *p* < 0.001) and were less likely to have experienced any IPV (28.6% versus 33.6%, *p* < 0.001) than included mothers. Mothers who were selected for the IPV module but excluded from the analysis because of missing data on exposure to IPV (there were no missing data on exclusive breastfeeding in the first 6 months) were less likely to exclusively breastfeed their infants in the first 6 months than included mothers (37.6% versus 45.3%, *p* < 0.001).

The mean age of mothers was 27.5 years. The proportion of mothers who had no formal education was 31.1%, and the proportion of mothers living in the lowest household wealth quintile was 25.4%. The prevalence of any lifetime exposure to IPV among mothers was 33.3% (27.6% for physical violence, 8.4% for sexual violence, and 16.4% for emotional violence). Breastfeeding was initiated within 1 hour of birth in 54.2% of newborns, and 44.4% of infants under 6 months were exclusively breastfed.

**Fig 2:**
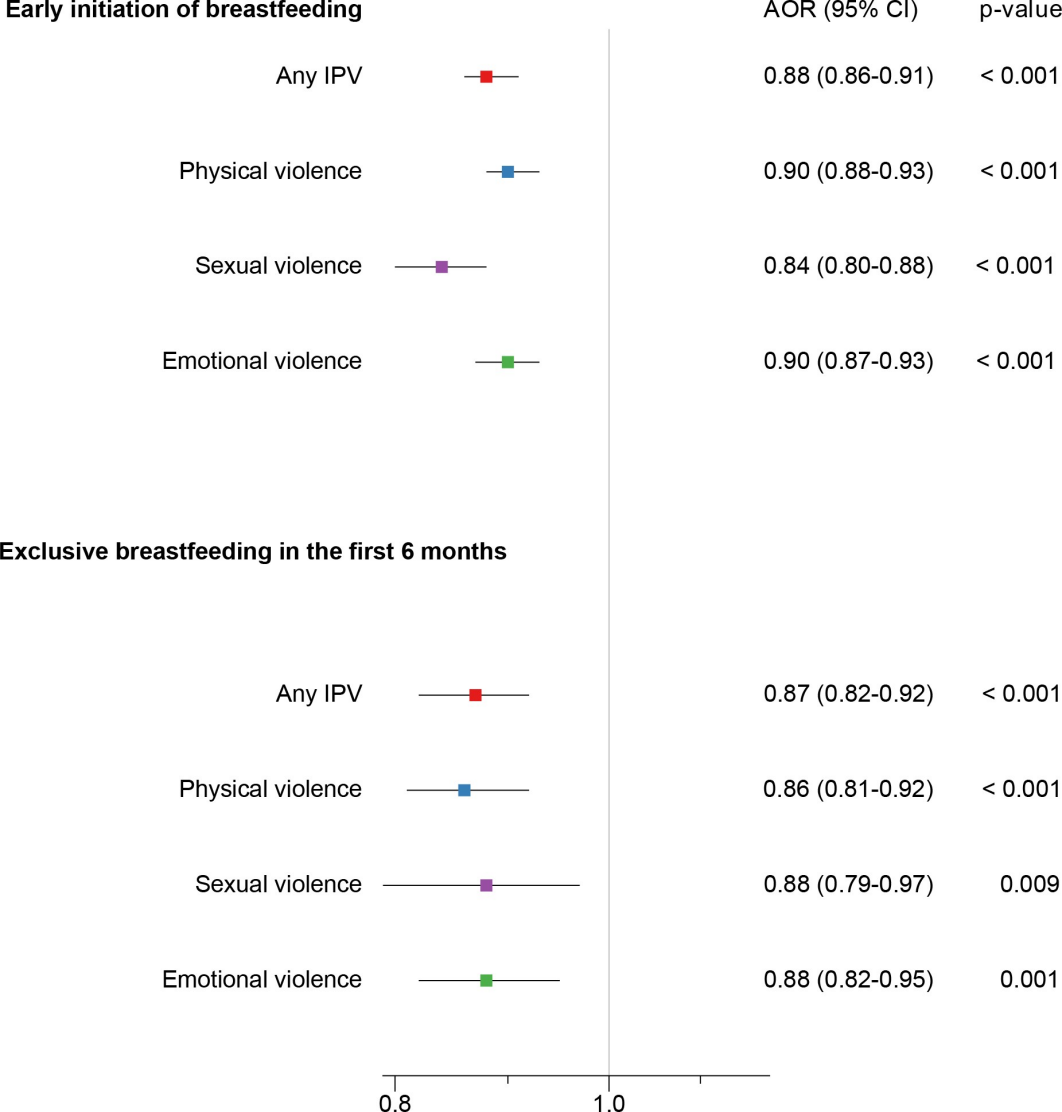
Association of mother’s exposure to different types of IPV with early initiation of breastfeeding and exclusive breastfeeding in the first 6 months. AOR, adjusted odds ratio; CI, confidence interval; IPV, intimate partner violence.

Fig 2 shows the adjusted associations between maternal exposure to IPV and early breastfeeding initiation. Overall, compared to mothers not exposed to any IPV, mothers exposed to any IPV were less likely to initiate breastfeeding early (AOR: 0.88 [95% CI 0.86–0.91], *p* < 0.001). Similar associations were found for maternal exposure to physical (AOR: 0.90 [95% CI 0.88–0.93], *p* = 0.001), sexual (AOR: 0.84 [95% CI 0.80–0.88], *p* = 0.001), and emotional violence (AOR: 0.90 [95% CI 0.87–0.93], *p* < 0.001). Compared to mothers not exposed to any IPV, mothers exposed to any IPV were also less likely to breastfeed their infants exclusively in the first 6 months (AOR: 0.87 [95% CI 0.82–0.92], *p* < 0.001). Similar associations were found for mothers exposed to physical (AOR: 0.86 [95% CI 0.81–0.92], *p* < 0.001), sexual (AOR: 0.88 [95% CI 0.79–0.97], *p* = 0.009), and emotional violence (AOR: 0.88 [95% CI 0.82–0.95], *p* < 0.001).

**Fig 3:**
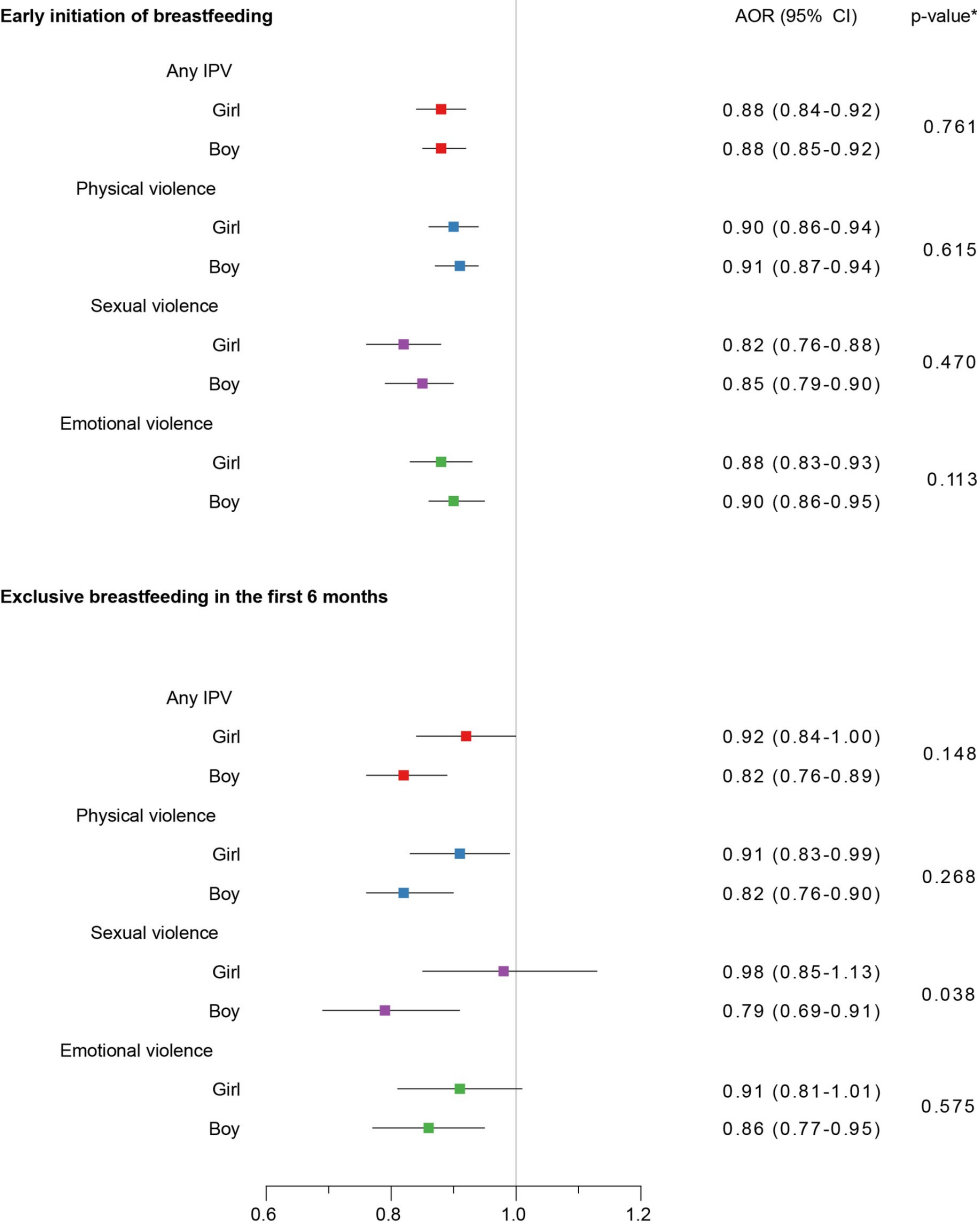
Association of mother’s exposure to different types of IPV with early initiation of breastfeeding and exclusive breastfeeding in the first 6 months—by infant sex. *p*-Values are for the interaction effect of IPV × sex in the model containing the main effects and this interaction. AOR, adjusted odds ratio; CI, confidence interval; IPV, intimate partner violence.

For subgroups defined by infant’s sex, both male and female infants belonging to mothers exposed to each type of IPV or any IPV were less likely to have initiated breastfeeding within the first hour of birth (Fig 3). Similarly, both male and female infants belonging to mothers exposed to each type of IPV (except sexual violence) or any IPV were less likely to have been exclusively breastfed in the first 6 months (Fig 3). Male infants compared to female infants were less likely to be exclusively breastfed if their mothers were exposed to sexual violence (*p* = 0.038).

**Fig 4:**
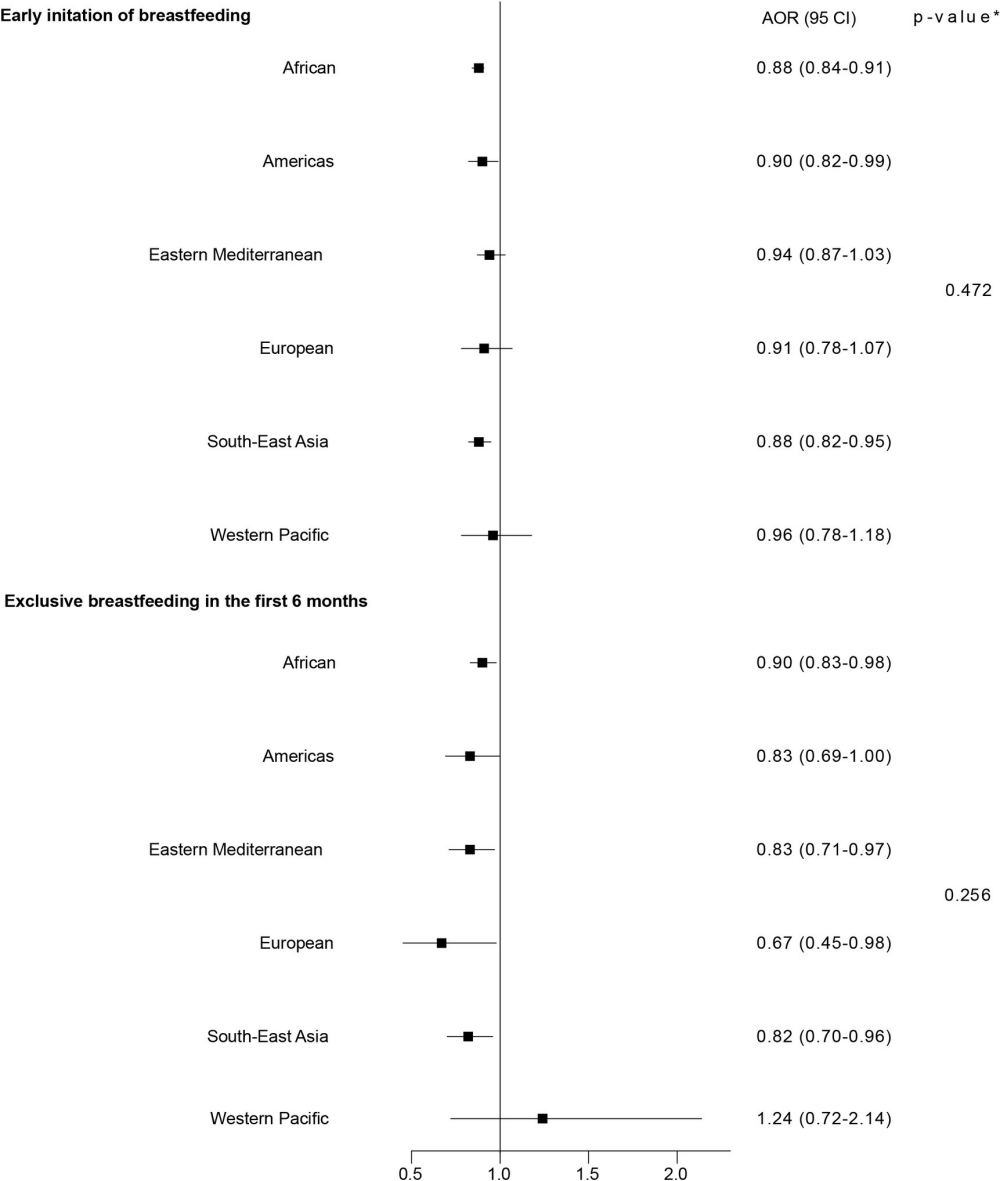
Association of mother’s exposure to any IPV with early initiation of breastfeeding and exclusive breastfeeding in the first 6 months—by WHO region. *p*-Values are for the interaction across WHO regions. AOR, adjusted odds ratio; CI, confidence interval; IPV, intimate partner violence.

For subgroups defined by WHO region, there were no significant differences in estimates across regions (Fig 4; S2 and S3 Tables).

In models where we simultaneously adjusted for all 3 types of IPV (i.e., physical, sexual, and emotional violence), exposure to each type of IPV was independently associated with a decreased likelihood of mothers’ initiating breastfeeding within 1 hour of birth. Only exposure to physical violence was independently associated with a decreased likelihood of mothers’ exclusively breastfeeding their infants under 6 months (S4 Table).

## Discussion

Using the most recently available nationally representative DHS data of mothers and children from 51 LMICs, we observed that mothers exposed to IPV were less likely to adopt recommended breastfeeding practices including initiating breastfeeding within the first hour of birth and breastfeeding exclusively in the first 6 months.

A previous systematic review of 12 studies on IPV and breastfeeding practices suggested that maternal exposure to IPV was associated with suboptimal breastfeeding practices [12]. Only 50.0% (6/12) of the studies were conducted in LMICs, and out of those studies, 66.7% (4/6) were from the same 2 countries. The most widely adopted scale for measuring IPV—the CTS2 was employed to measure exposure to IPV in the majority of the studies in LMICs (4/6); however, only 33.3% (2/6) of studies measured mothers’ exposure to emotional violence. In our study, we examined 51 LMICs, all country surveys used CTS2 to assess exposure to IPV, and exposure to emotional violence was measured in 86.3% (44/51) of countries. We examined both specific types of IPV (physical, sexual, and emotional) separately and any IPV. This is because in some relationships only 1 type of violence is present, but women can also be subjected to more than 1 type at a time [21]. Another important limitation of the systematic review was the fact that early initiation of breastfeeding was assessed in only 33.3% (2/6) of the studies in LMICs. In our study, all country surveys measured early initiation of breastfeeding.

Exposure to all forms of violence may influence a mother’s choice and ability regarding breastfeeding her infant. Overall, our analyses showed broadly consistent findings of maternal exposure to IPV increasing the likelihood of not initiating breastfeeding early and of not breastfeeding exclusively in the first 6 months. Male infants compared to female infants were less likely to be exclusively breastfed in the first 6 months if their mothers were exposed to sexual violence. However, this difference may have arisen due to chance. Further research would be needed to elucidate the sex differences in breastfeeding practices among mothers exposed to sexual violence. When we compared the different types of IPV exposure simultaneously, all types of IPV were independently negatively associated with early initiation of breastfeeding. However, only mothers exposed to physical violence were less likely to exclusively breastfeed their infants under 6 months. One interpretation of this result is that some barriers related to exclusive breastfeeding are more prevalent in mothers that have been exposed to physical violence.

Several mechanisms might explain the association between maternal exposure to IPV and suboptimal breastfeeding practices in LMICs. First, good prenatal and delivery care in LMICs has been suggested to significantly increase early breastfeeding initiation and the duration of exclusive breastfeeding in the first 6 months of life [22]. Women who experience IPV are reported to have social problems, lack of family support, including restricted access to services, strained relationships with health providers and employers, and isolation from social networks [23]. They are also reported to have different forms of physical, mental, and emotional health impairments (such as trauma, walking problems, chronic pain, stress, depression, anxiety, dizziness, and functional disorders). This all can lead to mothers’ initiating prenatal care later or receiving inadequate or no prenatal care [24–26]. Second, previous research suggests exposure to IPV is associated with depressive symptoms [27]. Mothers who are depressed are reported to be less likely to sustain breastfeeding [28]. Finally, if a mother’s self-esteem or confidence is lowered by exposure to IPV [29], then she may be less likely to start and maintain breastfeeding [30,31].

Several limitations, however, should be considered when interpreting our findings. First, the datasets we used are cross-sectional. While it is possible that IPV was the result of breastfeeding, such reverse causality seems relatively unlikely. Further analysis with longitudinal data is required to establish a temporal relation between maternal exposure to IPV and breastfeeding practices. The use of cross-sectional data also makes it challenging to exclude the possibility of residual confounding. Although we controlled for several potentially confounding variables based on the literature, there may have been confounding by unknown factors or imprecisely measured confounding variables such as household wealth. Second, DHS uses WHO ethical and safety protocols for research on IPV [32]. However, despite these protocols a proportion of women may not feel confident reporting IPV due to fear related to the consequences of disclosure or stigma [5,33], and so IPV may be subject to underreporting. Underreporting of mothers’ exposure to IPV experiences [34] would attenuate associations with early initiation of breastfeeding and exclusive breastfeeding towards the null, if exposure misclassification is independent of these outcomes. Third, mothers excluded from the analysis because of missing data either on early initiation of breastfeeding or IPV were less likely to initiate early breastfeeding and were less likely to have experienced any IPV than included mothers. Mothers who had been excluded from the analysis because of missing data on exposure to IPV were less likely to exclusively breastfeed their infants in the first 6 months than included mothers. The magnitude of these differences were relatively small.

Despite these limitations, our study has several strengths. First, our estimates for the associations between maternal exposure to IPV and breastfeeding practices are based on a relatively large and diverse sample of mother–infant dyads from 51 nationally representative household surveys that followed standardised procedures for the selection of women, with uniformity of information based on applying the same questions in all countries. Second, DHS also includes the CTS2, which is a validated and widely used questionnaire across countries to assess violence among intimate partners [20]. Finally, we examined maternal exposure to emotional violence and breastfeeding practices. Research has predominantly focused on maternal exposure to physical and/or sexual violence, despite emotional violence being recognised as an important component of women’s experiences of IPV [35]. Our study highlights the negative association of mother’s exposure to emotional violence with early initiation of breastfeeding irrespective of whether it is accompanied by physical or sexual violence.

IPV is a significant public health problem in LMICs, as well as a fundamental violation of women’s human rights [1]. Globally, the prevalence is highest in LMICs, particularly in the WHO African, Eastern Mediterranean, and South-East Asia Regions, where approximately 37% of ever-partnered women reported having experienced IPV at some point in their lives [3]. Due to insufficient evidence [36], the WHO does not recommend universal screening for IPV in women attending healthcare. However, the lack of evidence should not prevent healthcare professionals in LMICs from driving transformations in the healthcare response for IPV, considering the severity of the burden of experiencing IPV [33]. Targeted screening of women in the antenatal setting is crucial to identify women who have experienced or are experiencing IPV, connect them to essential services, and support them to initiate breastfeeding early and exclusively for 6 months. In the US, routine IPV screening and counselling is a core women’s preventive service under the Affordable Care Act [37], and IPV screening is recommended for women of reproductive age by the US Preventive Services Task Force [38]. Screening alone, without leveraging the entire healthcare environment, may not necessarily improve breastfeeding practices. In the US, the healthcare organisation Kaiser Permanente showed that a systems-level model can lead to substantial improvement in the healthcare system response to IPV. Its model included visible messaging for patients throughout the healthcare setting; routine private clinician inquiry, brief intervention, and referral; services including safety planning, triage for mental health needs, and follow-up; partnerships with IPV advocacy organisations; and local leadership and oversight [39]. Such models could be adopted and customised for women in antenatal settings in LMICs [40].

## Conclusion

Maternal exposure to any form of IPV (physical, sexual, or emotional violence) is associated with suboptimal breastfeeding practices in LMICs. Our findings may inform the argument for targeted screening of women in the antenatal setting who have experienced or are experiencing IPV, and the provision of services to support recommended breastfeeding practices.

## Supporting information

S1 STROBE Checklist (DOC)

S1 Table List of countries included in the analysis. (DOCX)

S2 Table Association of maternal exposure to different types of IPV with early initiation of breastfeeding. (DOCX)

S3 Table Association of maternal exposure to different types of IPV with exclusive breastfeeding in the first 6 months. (DOCX)

S4 Table Mutually adjusted association of maternal exposure to different types of IPV with early initiation of breastfeeding and exclusive breastfeeding in the first 6 months. (DOCX)

S1 Text Prospective analysis plan. (DOCX)

## Abbreviations

AOR: adjusted odds ratio
CTS2: Revised Conflict Tactics Scales
DHS: Demographic and Health Surveys
IPV: intimate partner violence
LMICs: low- and middle-income countries
WHO: World Health Organization
