# Supplementary material for: Maternal exposure to intimate partner violence and breastfeeding practices in 51 low-income and middle-income countries: A population-based cross-sectional study
Source: PLoS Med. 2019 Oct 1;16(10):e1002921. doi: 10.1371/journal.pmed.1002921 (PMC6771984; doi:10.1371/journal.pmed.1002921)
Supplement: S1 STROBE Checklist — (DOC) [file pmed.1002921.s001.doc]

**S2 Text**. STROBE Checklist

|  | Item No | Recommendation |
| --- | --- | --- |
| **Title and abstract** | 1 | (*a*) Indicate the study’s design with a commonly used term in the title or the abstract  **Abstract Paragraph 2 (Methods and findings): “We assessed population-based cross-sectional Demographic and Health Surveys (DHS) from 51 LMICs.”** |
|  |  | (*b*) Provide in the abstract an informative and balanced summary of what was done and what was found  **Abstract: Paragraph 1-3** |
| Introduction | | |
| Background/rationale | 2 | Explain the scientific background and rationale for the investigation being reported  **Introduction page 3 paragraphs 2-3** |
| Objectives | 3 | State specific objectives, including any prespecified hypotheses  **Introduction page 6 Paragraph 1: “…we therefore examined the association between maternal exposure to IPV with early initiation of breastfeeding and exclusive breastfeeding in the first six months in 51 LMICs.”** |
| Methods | | |
| Study design | 4 | Present key elements of study design early in the paper  **Methods “Study design and participants” (page 6 paragraph 3)** |
| Setting | 5 | Describe the setting, locations, and relevant dates, including periods of recruitment, exposure, follow-up, and data collection  **Methods page 6 paragraph 3**  **S1 Table** |
| Participants | 6 | (*a*) Give the eligibility criteria, and the sources and methods of selection of participants  **Figure 1**  **Results page 6 paragraph 3 and page 11 paragraph 2** |
| Variables | 7 | Clearly define all outcomes, exposures, predictors, potential confounders, and effect modifiers. Give diagnostic criteria, if applicable  **Methods pages 7-9: “Measure of maternal exposure to IPV”, “Breastfeeding practices” “A priori confounding variables”** |
| Data sources/ measurement | 8* | For each variable of interest, give sources of data and details of methods of assessment (measurement). Describe comparability of assessment methods if there is more than one group  **Methods pages 7-9: “Measure of maternal exposure to IPV”**  **” “Breastfeeding practices” “A priori confounding variables”** |
| Bias | 9 | Describe any efforts to address potential sources of bias  **Methods Statistical analysis (page 10 paragraph 2)** |
| Study size | 10 | Explain how the study size was arrived at  **Figure 1** |
| Quantitative variables | 11 | Explain how quantitative variables were handled in the analyses. If applicable, describe which groupings were chosen and why  **Methods: “Measure of IPV” (pages 7-8); “Breastfeeding practices” (pages 7); “A priori confounding variables” (page 9 paragraph 1)** |
| Statistical methods | 12 | (*a*) Describe all statistical methods, including those used to control for confounding  **Methods Statistical analysis (page 9 paragraph 2)** |
|  |  | (*b*) Describe any methods used to examine subgroups and interactions  **Methods Statistical analysis (page 10 paragraph 1-2)** |
|  |  | (*c*) Explain how missing data were addressed  **Results pages 11-12** |
|  |  | (*d*) If applicable, describe analytical methods taking account of sampling strategy  **Methods Statistical analysis (page 9-10)** |
|  |  | (*e*) Describe any sensitivity analyses |
| Results | | |
| Participants | 13* | (a) Report numbers of individuals at each stage of study—eg numbers potentially eligible, examined for eligibility, confirmed eligible, included in the study, completing follow-up, and analysed  **Figure 1**  **Results page 11-12** |
|  |  | (b) Give reasons for non-participation at each stage  **Figure 1** |
|  |  | (c) Consider use of a flow diagram  **Figure 1** |
| Descriptive data | 14* | (a) Give characteristics of study participants (eg demographic, clinical, social) and information on exposures and potential confounders  **Results page 11 paragraph 1 and page 12 paragraph 2** |
|  |  | (b) Indicate number of participants with missing data for each variable of interest  **Results pages 11 paragraph 2** |
| Outcome data | 15* | Report numbers of outcome events or summary measures  **Results page 12 paragraph 2** |
| Main results | 16 | (*a*) Give unadjusted estimates and, if applicable, confounder-adjusted estimates and their precision (eg, 95% confidence interval). Make clear which confounders were adjusted for and why they were included  **Statistical analysis (page 9 paragraph 1)**  **Figures 2-4** |
|  |  | (*b*) Report category boundaries when continuous variables were categorized |
|  |  | (*c*) If relevant, consider translating estimates of relative risk into absolute risk for a meaningful time period  **NA** |
| Other analyses | 17 | Report other analyses done—eg analyses of subgroups and interactions, and sensitivity analyses  **Figures 3-4** |
| Discussion | | |
| Key results | 18 | Summarise key results with reference to study objectives  **Discussion page 14 paragraph 1** |
| Limitations | 19 | Discuss limitations of the study, taking into account sources of potential bias or imprecision. Discuss both direction and magnitude of any potential bias  **Discussion page 16 paragraph 2** |
| Interpretation | 20 | Give a cautious overall interpretation of results considering objectives, limitations, multiplicity of analyses, results from similar studies, and other relevant evidence  **Discussion page 17 paragraph 2**  **Conclusion page 18 paragraph 2** |
| Generalisability | 21 | Discuss the generalisability (external validity) of the study results  **Discussion page 17 paragraph 1** |
| Other information | | |
| Funding | 22 | Give the source of funding and the role of the funders for the present study and, if applicable, for the original study on which the present article is based  **NA** |
