# Supplementary material for: Maternal exposure to intimate partner violence and breastfeeding practices in 51 low-income and middle-income countries: A population-based cross-sectional study
Source: PLoS Med. 2019 Oct 1;16(10):e1002921. doi: 10.1371/journal.pmed.1002921 (PMC6771984; doi:10.1371/journal.pmed.1002921)
Supplement: S1 Table — (DOCX) [file pmed.1002921.s002.docx]

**S1 Table. List of countries included in the analysis**

| Country | Year of survey | n |
| --- | --- | --- |
| Afghanistan | 2015 | 11,281 |
| Angola | 2015-2016 | 5,544 |
| Armenia | 2015-16 | 669 |
| Azerbaijan | 2006 | 842 |
| Bangladesh | 2007 | 2,228 |
| Burkina Faso | 2010 | 5,602 |
| Burundi | 2016-17 | 5,035 |
| Cambodia | 2014 | 2,815 |
| Cameroon | 2011 | 4,410 |
| Chad | 2014-2015 | 6,226 |
| Colombia | 2015 | 4,291 |
| Comoros | 2012 | 1,196 |
| Cote d'Ivoire | 2011-12 | 2,920 |
| Democratic Republic of Congo | 2013-2014 | 6,925 |
| Dominican Republic | 2013 | 1,355 |
| Egypt | 2014 | 6,321 |
| Ethiopia | 2016 | 3,861 |
| Gabon | 2018 | 2,350 |
| Ghana | 2008 | 1,175 |
| Guatemala | 2014-15 | 4,684 |
| Haiti | 2016 | 2,311 |
| Honduras | 2011-12 | 4,321 |
| India | 2015-16 | 94,388 |
| Jordan | 2012 | 3,633 |
| Kenya | 2014 | 7,602 |
| Kyrgyz Republic | 2012 | 1,764 |
| Liberia | 2007 | 2,023 |
| Malawi | 2015-16 | 6,243 |
| Maldives | 2016-2017 | 1,115 |
| Mali | 2012-13 | 3,794 |
| Moldova | 2005 | 6,243 |
| Mozambique | 2015 | 4,324 |
| Myanmar | 2015-16 | 1,807 |
| Namibia | 2013 | 1,828 |
| Nepal | 2016 | 1,878 |
| Nigeria | 2013 | 11,655 |
| Pakistan | 2012-2013 | 4,433 |
| Peru | 2012 | 3,556 |
| Philippines | 2017 | 3,703 |
| Rwanda | 2014-15 | 3,057 |
| Sao Tome and Principe | 2008-09 | 755 |
| Sierra Leone | 2013 | 4,203 |
| South Africa | 2016 | 1,195 |
| Tajikistan | 2017 | 2,219 |
| Tanzania | 2016 | 3,954 |
| Timor Leste | 2009-10 | 3,530 |
| Togo | 2013-14 | 2,673 |
| Uganda | 2016 | 5,577 |
| Ukraine | 2007 | 436 |
| Zambia | 2013-14 | 4,911 |
| Zimbabwe | 2015 | 2,185 |
