# Supplementary material for: Maternal exposure to intimate partner violence and breastfeeding practices in 51 low-income and middle-income countries: A population-based cross-sectional study
Source: PLoS Med. 2019 Oct 1;16(10):e1002921. doi: 10.1371/journal.pmed.1002921 (PMC6771984; doi:10.1371/journal.pmed.1002921)
Supplement: S2 Table — (DOCX) [file pmed.1002921.s003.docx]

**S2 Table. Association between maternal exposure to different types of IPV with early initiation of breastfeeding**

|  | Physical violence | Sexual violence | Emotional violence |
| --- | --- | --- | --- |
| WHO region | AOR (95% CI) | AOR (95% CI) | AOR (95% CI) |
| African | 0.91 (0.88-0.95) | 0.84 (0.79-0.89) | 0.90 (0.86-0.95) |
| Americas | 0.89 (0.80-0.99) | 0.98 (0.80-1.20) | 0.89 (0.79-1.01) |
| Eastern Mediterranean | 0.94 (0.86-1.02) | 0.78 (0.66-0.93) | 0.92 (0.84-1.01) |
| European | 0.93 (0.78-1.10) | 0.90 (0.57-1.40) | 0.99 (0.78-1.25) |
| South-East Asia | 0.88 (0.82-0.96) | 0.87 (0.75-1.00) | 0.88 (0.78-1.00) |
| Western Pacific | 0.95 (0.75-1.21) | 0.70 (0.48-1.03) | 0.94 (0.72-1.22) |
|  |  |  |  |
| p-value * | 0.718 | 0.821 | 0.907 |

AOR= adjusted odds ratio; CI= confidence intervals

Adjusted for mother’s age, mother’s level of education, household wealth, rural or urban residence, child’s age and child’s sex

*p-value for interaction across WHO regions.
