# Supplementary material for: Maternal exposure to intimate partner violence and breastfeeding practices in 51 low-income and middle-income countries: A population-based cross-sectional study
Source: PLoS Med. 2019 Oct 1;16(10):e1002921. doi: 10.1371/journal.pmed.1002921 (PMC6771984; doi:10.1371/journal.pmed.1002921)
Supplement: S3 Table — (DOCX) [file pmed.1002921.s004.docx]

**S3 Table. Association between maternal exposure to different types of IPV with exclusive breastfeeding in the first six months**

|  | Physical violence | Sexual violence | Emotional violence |
| --- | --- | --- | --- |
| WHO region | AOR (95% CI) | AOR (95% CI) | AOR (95% CI) |
| African | 0.89 (0.82-0.97) | 0.90 (0.79-1.01) | 0.89 (0.81-0.98) |
| Americas | 0.83 (0.68-1.01) | 0.85 (0.58-1.25) | 0.78 (0.60-1.02) |
| Eastern Mediterranean | 0.85 (0.72-1.00) | 0.76 (0.55-1.03) | 0.89 (0.75-1.07) |
| European | 0.75 (0.50-1.12) | 0.57 (0.16-2.03) | 0.66 (0.36-1.22) |
| South-East Asia | 0.77 (0.65-0.91) | 0.95 (0.71-1.27) | 0.94 (0.73-1.21) |
| Western Pacific | 1.00 (0.52-1.90) | 1.29 (0.49-3.36) | 1.66 (0.72-3.83) |
|  |  |  |  |
| p value* | 0.189 | 0.885 | 0.906 |

AOR= adjusted odds ratio; CI= confidence intervals

Adjusted for mother’s age, mother’s level of education, household wealth, rural or urban residence, child’s age and child’s sex

*p-value for interaction across WHO regions.
