# Supplementary material for: Maternal exposure to intimate partner violence and breastfeeding practices in 51 low-income and middle-income countries: A population-based cross-sectional study
Source: PLoS Med. 2019 Oct 1;16(10):e1002921. doi: 10.1371/journal.pmed.1002921 (PMC6771984; doi:10.1371/journal.pmed.1002921)
Supplement: S4 Table — (DOCX) [file pmed.1002921.s005.docx]

**S4 Table. Mutually adjusted association between maternal exposure to different types of IPV with early initiation of breastfeeding and exclusive breastfeeding in the first six months**

|  | Early initiation of breastfeeding | p-value | Exclusive breastfeeding in the first six months | p-value |
| --- | --- | --- | --- | --- |
|  | AOR (95% CI) |  | AOR (95% CI) |  |
| IPV type |  |  |  |  |
| Physical violence | 0.94 (0.91-0.98) | 0.002 | 0.89 (0.83-0.96) | 0.002 |
| Sexual violence | 0.87 (0.83-0.92) | <0.001 | 0.94 (0.84-1.05) | 0.256 |
| Emotional violence | 0.95 (0.91-0.99) | 0.018 | 0.95 (0.87-1.03) | 0.211 |

AOR= adjusted odds ratio; CI= confidence intervals

Adjusted for mother’s age, mother’s level of education, household wealth, rural or urban residence, child’s age and child’s sex
