# Supplementary material for: Maternal exposure to intimate partner violence and breastfeeding practices in 51 low-income and middle-income countries: A population-based cross-sectional study
Source: PLoS Med. 2019 Oct 1;16(10):e1002921. doi: 10.1371/journal.pmed.1002921 (PMC6771984; doi:10.1371/journal.pmed.1002921)
Supplement: S1 Text — (DOCX) [file pmed.1002921.s006.docx]

**S1 Text**. Prospective analysis plan

Maternal exposure to intimate partner violence and breastfeeding practices in 51 low-income and middle-income countries

**Research Objectives**

1.

1. Examine the association between maternal exposure to IPV (physical violence, sexual violence, and emotional violence, and any type of IPV, respectively) with early initiation of breastfeeding. Control for mother’s age, mother’s level of education, household wealth, rural or urban residence, child’s age and child’s sex.
2. Stratify association by sex and WHO region (African, Americas, Eastern Mediterranean, European, South-East Asia and Western Pacific), respectively.

2.

1. Examine the association between maternal exposure to IPV (physical violence, sexual violence, and emotional violence, and any type of IPV, respectively) with exclusive breastfeeding. Control for mother’s age, mother’s level of education, household wealth, rural or urban residence, child’s age and child’s sex.
2. Stratify association by sex and WHO region (African, Americas, Eastern Mediterranean, European, South-East Asia and Western Pacific), respectively.

3. Examine the association between maternal exposure to each type of IPV (physical violence, sexual violence, and emotional violence) simultaneously with early initiation of breastfeeding. Control for mother’s age, mother’s level of education, household wealth, rural or urban residence, child’s age and child’s sex.

4. Examine the association between maternal exposure to each type of IPV (physical violence, sexual violence, and emotional violence) simultaneously with exclusive breastfeeding. Control for mother’s age, mother’s level of education, household wealth, rural or urban residence, child’s age and child’s sex.

4. Test for a cross-level interaction effect between wealth index and country.
